# Reports of violence against transvestites and transsexuals: a time series study, Brazil, 2015-2022

**DOI:** 10.1590/S2237-96222025v34e20240350.en

**Published:** 2025-08-04

**Authors:** Everton Barroso Rios, Samuel Trezena Costa, Mara Daisy Alves Ribeiro, Luciana Colares Maia, Simone de Melo Costa

**Affiliations:** 1 Universidade Estadual de Montes Claros, Departamento de Odontologia, Programa de Pós-Graduação em Cuidado Primário em Saúde, Montes Claros, MG, Brazil; 2 Universidade Estadual de Montes Claros, Departamento de Odontologia, Programa de Pós-Graduação em Ciências da Saúde, Montes Claros, MG, Brazil

**Keywords:** Sexual and Gender Minorities, Violence, Health Information Systems, Health Vulnerability, Time Series Studies, Minorías Sexuales y de Género, Violencia, Sistemas de Información en Salud, Vulnerabilidad en Salud, Estudios de Series Temporales

## Abstract

**Objective:**

To characterize reports of violence against transvestites and transsexuals in Brazil from 2015 to 2022.

**Methods:**

This is a descriptive time-series study. Secondary data was obtained from the Interpersonal and Self-Inflicted Violence Surveillance System, part of the Notifiable Health Conditions Information System. Cases of victims identified as “transvestites” and “transsexual women or men” were selected from the reports for 2015 to 2022. The reports were characterized according to the profiles of the victim and the perpetrator; characteristics of the violence; temporal trend for transvestite and transsexual victims; and trend by Brazilian macro-region. A descriptive analysis of absolute and relative frequencies was performed.

**Results:**

We selected 37,104 reports of interpersonal/self-inflicted violence against transvestites and transsexuals in the study period. Transsexual women (66.1%), heterosexuals (54.1%), adults (49.5%), and individuals of mixed race/skin color (44.5%) were the groups that predominantly suffered some type of violent episode. The percentage increase in the period was greater among transvestites (+182.0%). As for the time series by Brazilian macro-regions, an increasing trend in the number of reports was identified. The Southeast and Midwest regions showed an increase of 155.0% in reports from 2015 to 2022, while there was an 81.0% increase in the Southern region, 72.0% in the North and 69.0% in the Northeast.

**Conclusion:**

Over the period, more cases were found for transsexual women victims. However, from 2015 to 2022, a greater percentage increase in cases was identified for transvestites. More cases were identified in the Southeast and Northeast regions, but the Midwest region is equal to the Southeast in terms of the percentage increase in reports from 2015 to 2022.

## Resumen

**Objetivo:**

Caracterizar las notificaciones de violencia contra travestis y transexuales en Brasil en el período de 2015 a 2022.

**Métodos:**

Estudio descriptivo de series temporales. Los datos secundarios provienen del Sistema de Vigilancia de Violencia Interpersonal y Autoinfligida del Sistema de Información de Enfermedades de Notificación Obligatoria. Se seleccionaron casos de víctimas identificadas como “travestis” y “mujeres u hombres transexuales” en las notificaciones de 2015 a 2022. Las notificaciones se caracterizaron por el perfil de la víctima y del agresor; características de la violencia; tendencia temporal para víctimas travestis y transexuales; y tendencia por macrorregión brasileña. Se realizó un análisis descriptivo de frecuencias absolutas y relativas.

**Resultados:**

Se seleccionaron 37.104 notificaciones de violencia interpersonal/autoinfligida contra personas travestis y transexuales en el periodo del estudio. Las mujeres transexuales (66,1%), heterosexuales (54,1%), personas adultas (49,5%) y personas de raza/color de piel mestizos (44,5%) fueron los grupos que predominantemente sufrieron algún tipo de episodio violento. El incremento porcentual en el período fue mayor entre las travestis (+182,0%). Respecto a la serie temporal por macroregiones brasileñas, se identificó una tendencia creciente en el número de notificaciones. Las regiones Sudeste y Centro-Oeste presentaron un incremento de notificaciones de 155,0% de 2015 a 2022, la región Sur tuvo un incremento de 81,0%, la Norte, de 72,0% y la Nordeste, de 69,0%.

**Conclusión:**

Durante este período hubo más casos que involucraron a mujeres transexuales como víctimas. Sin embargo, de 2015 a 2022 se identificó un mayor incremento porcentual de casos de personas travestis. Se identificaron más casos en las regiones Sudeste y Nordeste, pero la región Centro-Oeste es igual a la Sudeste en términos del aumento porcentual de notificaciones de 2015 a 2022.

**Ethical aspects**

This research used public domain anonymized databases.

## Introduction

The term “gender” refers to a set of sociocultural behaviors established and delimited by binary logic to classify characteristics associated with male and female (1), while gender identity encompasses a set of values, impulses, and experiences acquired throughout life, legitimizing the different ways of existing. In this context, transgender people, both male and female, do not identify with the gender assigned at birth, but, throughout their lives, recognize themselves as transsexual men and women or transvestites (2).

It is noteworthy that, within the community of lesbian, gay, bisexual, transgender, queer, intersex, asexual people, and those of other sexualities and gender identities (LGBTQIA+, formerly known as LGBT) (3), transgender people are those who face the most prejudice and discrimination, especially in the family, social, and health contexts (4).

In terms of health, violence can be defined as an intentional action that, through physical force or power, causes injury, death, psychological harm, developmental disabilities and/or deprivation. Self-inflicted violence and interpersonal violence stand out among the classification of types of violence, (5) Both are included in the category harm caused by external causes and have a major impact on the health conditions and quality of life of the population. Therefore, the Ministry of Health recommends reporting all suspected or confirmed cases of interpersonal and self-inflicted violence (6).

However, it was only in October 2014 that the individual reporting form for interpersonal/self-inflicted violence, proposed by the Ministry of Health, received new fields to be filled out to identify incidents of violence against the LGBTQIA+ population. Information such as social name, sexual orientation, gender identity and reason for violence were added (6). Obtaining data from reported cases is essential for formulating public policies aimed at combating the various forms of violence. Due to stigmatization, structural prejudices and social heteronormativity, there are few studies that focus on assessing the health conditions and needs of the LGBTQIA+ population, which makes it difficult to infer the real magnitude of the conditions faced by these individuals (3).

Furthermore, the few studies that measure episodes of violence against the “queer” community present specific locations and short periods of time, and analyze community members as a whole (2,7-11). It is important to conduct specific studies for transgender people, who are marginalized individuals and are more likely to suffer episodes of violence, hate crimes, and health service deprivation (12). As such, the objective of this study was to characterize reports of violence against transvestites and transsexuals in Brazil from 2015 to 2022.

## Methods

### Design

This is a descriptive, time-series study, conducted according to the guidelines of the Reporting of Studies Conducted Using Observational Routinely-collected health Data (RECORD) Statement (13).

### Setting

When categorizing types of violence, self-inflicted violence and interpersonal violence stand out, and both have a major impact on the health conditions and quality of life of the population. Thus, the Ministry of Health recommends reporting all suspected or confirmed cases of violence (5).

### Participants

Reports of interpersonal/self-harm violence involving transsexual and transvestite people were considered eligible for the study.

### Variables

The variables selected are part of the interpersonal/self-inflicted violence reporting form, namely: Federative Units grouped by Brazilian regions (North, Northeast, Midwest, Southeast and South); race/skin color; age, categorized as adolescent (11-17 years old), young adult (18-24 years old), adult (25-59 years old) and elderly (60 years old or over) (16,17); gender identity; sexual orientation; place of occurrence; recurrence of violence; self-inflicted injury; reason for violence; type of violence; form of sexual violence; number of perpetrators involved; relationship/degree of kinship of the perpetrator with the victim; and year of occurrence of violence (2015 to 2022).

### Data sources and measurement 

The secondary data were obtained from the Interpersonal and Self-Inflicted Violence Surveillance System, part of the Notifiable Health Conditions Information System (*Sistema de Vigilância de Violência Interpessoal e Autoprovocada* / *Sistema de Informação de Agravos de Notificação* - VIVA/SINAN), maintained by the Information Technology Department of the Brazilian National Health System (DATASUS), which consolidates the reporting and investigation records of cases of diseases and health conditions for which reporting is compulsory (14).

### Study size

The time period for the data description was from 2015 to 2022. This period was selected due to the inclusion of the gender identity and sexual orientation identification fields on the compulsory reporting forms in 2014 (6). Data for the year 2023 were not included, as they were not fully available at the time of data collection for this research.

### Data access and cleaning methods

The data were accessed in January 2024 using the Tab for Windows (TabWin) application, available online for download on the DATASUS website (14). Files were imported and later aggregated into spreadsheets using Microsoft Office Excel 2013. In order to identify the study target population, cases in which the “gender identity” field was filled in with “not applicable” or “unknown”, or was left “blank” were excluded. Cases in which the victims were identified as “transvestites”, “transgender women” and “transgender men” were selected. After excluding inadequately completed reports, cases and fields filled in incorrectly or with divergent information were analyzed for inconsistency (15). Atypical cases were excluded based on descriptive analysis, with contingency tables and outliers (for example, individuals who were under one year old and transgender individuals of the male sex for whom the “pregnant” field was selected). Reports of victims under the age of 11 were also excluded.

### Statistical methods

The data were organized on Excel spreadsheets and checked by two trained researchers. They were subsequently entered into the Statistical Package for the Social Sciences 27.0, and descriptive analysis was conducted with absolute (n) and relative (%) frequencies. 

## Results

In the study period, 37,104 notifications of interpersonal/self-inflicted violence against transvestite and transsexual individuals were selected. Transsexual women (66.1%), heterosexuals (54.1%), adults (49.5%) and individuals of mixed race/skin color (44.5%) were the groups that predominantly suffered some type of violent episode, reported between 2015 and 2022 (Table 1).

**Table 1:** Profile of victims of interpersonal/self-inflicted violence against transvestites and transsexuals. Brazil, 2015-2022 (n=37,104)

Variable	n (%)
**Gender identity**	
Transvestite	5,241 (14.1)
Transsexual woman	24,522 (66.1)
Transsexual man	7,341 (19.8)
**Sexual orientation**	
Heterosexual	20,069 (54.1)
Homosexual	10,421 (28.1)
Bisexual	1,112 (3.0)
Unknown/left blank	5,502 (14.8)
**Age group** (years)	
Adolescent (11-17)	6,299 (17.0)
Young adult (18-24)	10,548 (28.4
Adult (25-59)	18,367 (49.5)
Elderly (60 or over)	1,890 (5.1)
**Race/skin color**	
White	14,148 (38.1)
Black	3,710 (10.0)
Asian	315 (0.8)
Mixed race	16,522 (44.5)
Indigenous	455 (1.2)
Unknown/left blank	1,956 (5.3)

**Figure 1:**
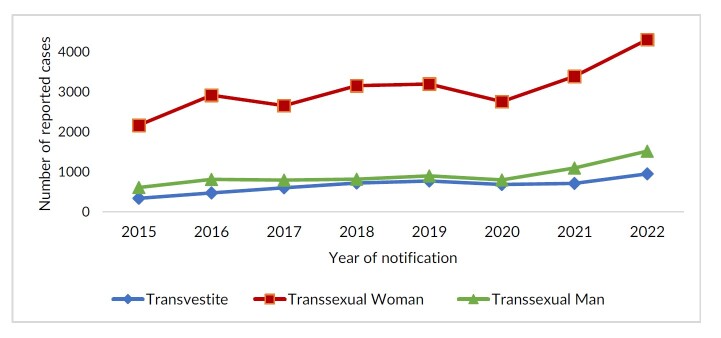
Temporal trend of reports of interpersonal/self-inflicted violence against transsexuals and transvestites. Brazil, 2015-2022 (n=37,104)

Reporting distribution in relation to time, according to gender identity, shows that the largest number of reports from 2015 to 2022 was for transsexual women (n=24,522). From 2018 to 2019, reports were seen to have a stationary trend (n=3,153 and n=3,195), falling in 2020 (n=2,752) and returning with a significant increase in 2022 (n=4,304), as shown in Figure 1. The percentage increase between 2015 and 2022 was greater among transvestites (+182.0%); being 99.0% for transsexual women and 149.0% for transsexual men. 

Most reported cases occurred at the victims’ residence (64.0%), and the most prevalent types of violence were physical violence (61.7%), self-inflicted violence (30.1%), followed by psychological/moral violence (22.9%). Of the reported cases, 40.7% were recurrent episodes. LGBTphobia was the reason for the violent episode in 6.2% of cases (Table 2).

**Table 2:** Characterization of violence against transvestites and transsexuals. Brazil, 2015-2022 (n=37,104)

Variable	n (%)
**Place of occurrence**	
Residence	23,748 (64.0)
Collective housing	592 (1.6)
School	512 (1.4)
Sports facility	140 (0.4)
Bar or similar	1,273 (3.4)
Public thoroughfare	6,780 (18.4)
Commerce/services	578 (1.6)
Industries/construction	80 (0.2)
Others	1,743 (4.7)
Unknown/left blank	1,658 (4.4)
**Recurring episode**	
Yes	15,107 (40.7)
No	17,359 (46.8)
Unknown/left blank	4,638 (11.5)
**Self-inflicted violence**	
Yes	11,176 (30.1)
No	24,134 (65.0)
Unknown/left blank	1,794 (4.9)
**Reason for violence**	
Sexism	3,092 (8.3)
Homophobia/lesbophobia/biphobia/transphobia	2,312 (6.2)
Racism	40 (0.1)
Religious intolerance	45 (0.1)
Xenophobia	39 (0.1)
Generational conflict	4,952 (13.3)
Street dweller	1,419 (3.8)
Disability	366 (1.0)
Other	10,257 (27.6)
Not applicable	14,134 (38.1)
**Type of violence**	
Physical	25,992 (61.7)
Psychological/moral	9,648 (22.9)
Torture	1,400 (3.3)
Sexual	3,396 (8.1)
Human trafficking	23 (0.1)
Financial/economic	557 (1.3)
Neglect/abandonment	993 (2.4)
Legal intervention	86 (0.2)
**Form of sexual violence**	
Sexual harassment	793 (22.8)
Rape	2,537 (73.1)
Sexual exploitation	141 (4.1)

The probable perpetrator of the incidents was the victim themself (29.9%), followed by spouse (15.7%) and acquaintance/friend (13.3%). Suspected alcohol use by the perpetrator was reported in 31.6% of the reports (Table 3).

**Table 3:** Profile of the probable perpetrator of violence against transvestites and transsexuals. Brazil, 2015-2022 (n=37,104)

Variables	n (%)
**Number involved**	
One	26,857 (72.4)
Two or more	8,010 (21.6)
Unknown/left blank	2,237 (6.0)
**Relationship/degree of kinship with the victim**	
The person themself	10,509 (29.9)
Father	986 (2.8)
Mother	910 (2.6)
Stepfather	479 (1.4)
Stepmother	57 (0.2)
Spouse	5,501 (15.7)
Ex-spouse	1,790 (5.1)
Boy/girlfriend	1,448 (4.1)
Ex-boy/girlfriend	634 (1.8)
Son/daughter	991 (2.8)
Brother/sister	1,091 (3.1)
Acquaintance/friend	4,687 (13.3)
Caregiver	101 (0.3)
Boss	136 (0.4)
Person with an institutional relationship	246 (0.7)
Police officer/law enforcement officer	350 (1.0)
Stranger	5,226 (14.9)

**Figure 2:**
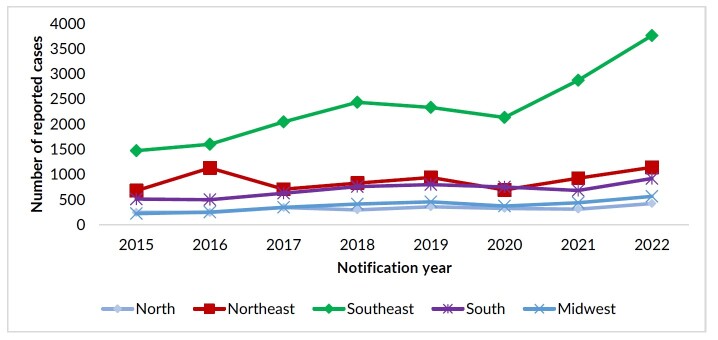
Temporal trend of reports of interpersonal/self-inflicted violence against transsexuals and transvestites by region. Brazil, 2015-2022 (n=37,104)

As for the time series by Brazilian regions, an increasing trend in the number of reports was identified in the period 2015-2022, with the highest number of cases in this period in the Southeast region (n=18,666), followed by the Northeast (n=7,052), South (n=5,549), Midwest (n=3,064) and North (n=2,548) (Figure 2). The Southeast and Midwest regions showed an increase of 155.0% in reports between 2015 and 2022; while there was an 81.0% increase in the Southern region, 72.0% in the North and 69.0% in the Northeast 

## Discussion

This research contributes to the Brazilian National Unified Health System (*Sistema Único de Saúde* - SUS) by presenting data on violence against transvestites and transsexuals in Brazil. An increase in reports of aggressions directed at transgender people and transvestites, registered on the SINAN, was observed in all regions of Brazil, mainly in the Southeast region. It is important to highlight that most of these reports were identified for transsexual women. This data emphasizes the finding that, when they diverge from established social norms, manifestations of estrangement in relation to expressions of sexuality and gender can trigger violent acts (9).

When trans women face exclusions due to their gender identity, whether in public or private environments, this amplifies the creation of marginalized scenarios, contributing to experiences of educational dropout and precarious socioeconomic conditions (18).

It was found that the age groups most affected by violence were young adults and adults themselves. The predominant ages described in the literature range from 25 to 59 years (2). However, for transgender people, occurrences among transsexual women were more common in all age categories from adolescence onwards, with a proportion of 37.0% among adolescents, 36.3% among elderly women and 31.8% among adult women. The victims are usually of mixed or White race/skin color (9). It is important to note that the description of race/skin color, on the compulsory reporting form, is self-reported by the victim (11,19).

We identified that the main settings in which aggressions against LGBTQIA+ individuals occurred were residences and public thoroughfares. In cases involving violence in private settings, it can be understood that the family or intimate unit does not act as a support and protection network, aggravating the effects of the harm caused by social discrimination experienced in public spaces. An ethnographic study with trans people, carried out in Santa Maria/Rio Grande do Sul, in 2012, highlighted that the home is the environment where violent behaviors of prejudice, discrimination and physical aggression manifest themselves early, sometimes resulting in eviction from the home (20,21).

The most prevalent types of violence were physical, psychological, and self-inflicted, in addition to sexual violence, classified as rape. In a cross-sectional study developed through analysis of reports of suspected or confirmed cases of interpersonal violence, from 2016 to 2020, in the city of São Paulo, the most recorded type of violence was physical, accounting for 76.3% (n=3,683), followed by psychological/moral violence, accounting for 32.6% (n=1,576), and sexual violence, accounting for 17.7% (n=854). Almost all reports related to transgender people had a history of psychological abuse, and more than 80.0% of these people had already been victims of physical or sexual violence (11,22).

It is also worth noting that sexual violence, when directed at people who identify as LGBTQIA+, can acquire a different connotation, being used as a form of punishment and humiliation for the victims, for being who they are. In this way, stigmatization and shame generate social exclusion that impacts, among other aspects, access to/seeking health care and the difficulty in accessing services (23). Psychological violence can be defined as attacks on the identity and other essential qualities of the individual, such as self-esteem, both by action and omission. From this perspective, we can relate it to the situations faced daily by transsexual people, in which their identities are ignored and not respected in various social spaces (24).

Almost one-third of the reports analyzed refer to self-inflicted violence. The LGBTQIA+ community in particular faces a series of overlapping types of violence, affecting health and contributing to suffering and illness (8). Evidence indicates that self-inflicted violence has high rates among members of the LGBTQIA+ community when compared to heterosexual individuals. It is worth noting that transsexual people face a high risk of suicide compared to the general population (2).

When analyzing the factors related to recurrence, sexual orientation and gender identity by age group and comparing them with the general population, it is found that young homosexual adults have an 87.0% higher risk and transsexual/transvestite adults a 66.0% higher risk of presenting self-inflicted violence (10). Considering that the residence was the place where most situations of violence occurred, the practice of self-inflicted injuries may be associated with the denial of people’s sexual orientation and gender identity within the context of family relationships (9).

This reality extends to other sectors, in which acceptance and employment opportunities are challenging, consequently highlighting the prevalence of informal work. As a result, many transsexual and transvestite people find themselves in work linked to prostitution, recognized as an environment conducive to various forms of violence (25). From this perspective, markers related to gender and sexual diversity have been recognized as components of the social determinants of the health and disease process, through the creation of the National Policy for Comprehensive LGBT Health, in view of the need for equity actions within the scope of the SUS for this population (9).

The lack of rights guaranteed to the transsexual population and the naturalized violence against this group tend to persist, requiring an ongoing active search for solutions to eradicate violence. It is urgent to continue with the social inclusion of trans people in institutional activities, through partnerships aimed at mitigating their everyday difficulties, such as the implementation of legislation to prevent violence, investigate and hold criminals accountable. Furthermore, violent and transphobic speech must be rejected, and groups that exclude trans persons and attempt to persecute them, whether through silencing or acts of violence, must be repudiated (26).

Among the reports of violence, missing information for certain variables stands out, which constitutes a limitation of this study. It should also be considered that the number of cases of violence against the group studied is actually greater than that presented, due to underreporting of occurrences. As a strength of this study, all valid cases for the target audience, transvestites and transsexuals, in Brazil, were considered, from the perspective of the eight-year time series.

Our analysis of the reports reveals an alarming scenario of violence against transvestites and transsexuals in Brazil, between 2015 and 2022. Predominantly, transsexual women were identified as the main victims, together with the high occurrence of episodes in residences. Repetition of episodes of violence occurred in almost half of the reports, and one of the reasons for violent acts was LGBTphobia, although self-inflicted violence was also frequent. Regarding the temporal trend of reports by the country’s macro-regions, higher numbers of cases were identified in the Southeast and Northeast regions; however, the Midwest region is equal to the Southeast with regard to the percentage increase in reports.

The data provided by this research can help the Brazilian State to understand the problem of LGBTphobia, as well as to support the importance of public policies in combating violence against the transsexual and transvestite population and in promoting acceptance and equity for this segment of the population.

## Data Availability

The database and the analysis codes used in this research are available at http://tabnet.datasus.gov.br/cgi/deftohtm.exe?sinannet/cnv/violebr.def.
