# Supplementary material for: Reports of violence against transvestites and transsexuals: a time series study, Brazil, 2015-2022
Source: Epidemiol Serv Saude. 2025 Aug 4;34:e20240350. doi: 10.1590/S2237-96222025v34e20240350.en (PMC12334237; doi:10.1590/S2237-96222025v34e20240350.en)
Supplement: Translation

# Translation: Notificações de violência contra travestis e transexuais: estudo de série temporal, Brasil, 2015-2022

**DOI:** 10.1590/S2237-96222025v34e20240350.pt

**Authors:** Everton Barroso Rios, Samuel Trezena Costa, Mara Daisy Alves Ribeiro, Luciana Colares Maia, Simone de Melo Costa

**Affiliations:** 1 Universidade Estadual de Montes Claros, Departamento de Odontologia, Programa de Pós-Graduação em Cuidado Primário em Saúde, Montes Claros, MG, Brazil; 2 Universidade Estadual de Montes Claros, Departamento de Odontologia, Programa de Pós-Graduação em Ciências da Saúde, Montes Claros, MG, Brazil

**Keywords:** Minorias Sexuais e de Gênero, Violência, Sistemas de Informação em Saúde, Vulnerabilidade em Saúde, Estudos de Séries Temporais

## Abstract

**Objetivo:**

Caracterizar as notificações de violência contra travestis e transexuais, no Brasil, no período de 2015 a 2022.

**Métodos:**

Estudo descritivo de série temporal. Os dados secundários são provenientes do Sistema de Vigilância de Violência Interpessoal e Autoprovocada do Sistema de Informação de Agravos de Notificação. Selecionaram-se casos de vítimas identificadas como “travestis” e “mulheres ou homens transexuais” nas notificações de 2015 a 2022. A caracterização das notificações deu-se pelo perfil da vítima e do agressor; características da violência; tendência temporal por vítimas travestis e transexuais; e tendência por macrorregião brasileira. Realizou-se análise descritiva das frequências absolutas e relativas.

**Resultados:**

No recorte temporal, selecionaram-se 37.104 notificações de violência interpessoal/autoprovocada contra indivíduos travestis e transexuais. Mulheres transexuais (66,1%), heterossexuais (54,1%), adultos (49,5%) e indivíduos de raça/cor da pele parda (44,5%) foram os grupos que predominantemente sofreram algum tipo de episódio violento. O aumento percentual no período foi maior entre travestis (+182,0%). Quanto à série temporal por macrorregiões brasileiras, foi identificada tendência crescente no número de notificações. As regiões Sudeste e Centro-Oeste apresentaram aumento de 155,0% das notificações de 2015 para 2022, a região Sul teve aumento de 81,0%, a Norte, de 72,0%, e a Nordeste, de 69,0%.

**Conclusão:**

Constatam-se no período mais casos para vítimas mulheres transexuais. No entanto, de 2015 para 2022, identifica-se maior aumento percentual de casos para indivíduos travestis. Identificaram-se mais casos nas regiões Sudeste e Nordeste, mas a região Centro-Oeste se iguala ao Sudeste no que diz respeito ao aumento percentual das notificações de 2015 para 2022.

**Aspectos éticos**

Esta pesquisa utilizou bancos de dados de domínio público e anonimizados.

## Introdução

O termo “gênero” faz referência a um conjunto de comportamentos socioculturais estabelecidos e delimitados pela lógica binária para classificar as características associadas ao masculino e feminino (1), sendo que a identidade de gênero abarca um conjunto de valores, impulsos e vivências adquiridos ao longo da vida, legitimando as diversas formas de existir. Nesse contexto, pessoas transgênero, tanto do sexo masculino quanto feminino, não se identificam com o gênero atribuído ao nascimento, mas, ao longo da vida, reconhecem-se como homens e mulheres transexuais ou travestis (2).

Destaca-se que, dentro da comunidade de lésbicas, gays, bissexuais, transgêneros, queers, intersexuais, assexuais e outras sexualidades e identificações de gênero (LGBTQIA+, sigla anteriormente denominada LGBT) (3), as pessoas transgêneros são as que enfrentam mais preconceito e discriminação, especialmente no âmbito familiar, social e na saúde (4). 

Para a saúde, a violência pode ser definida como uma ação intencional que, por meio da força física ou poder, promova lesão, morte, dano psicológico, deficiência de desenvolvimento e/ou privação. Na classificação dos tipos de violência, destacam-se a violência autoprovocada e a violência interpessoal (5). Ambas estão inseridas nos agravos provocados pelas causas externas e produzem grande impacto nas condições de saúde e qualidade de vida da população. Dessa maneira, o Ministério da Saúde recomenda notificar todo caso suspeito ou confirmado de violência interpessoal e autoprovocada (6). 

No entanto, somente em outubro de 2014, a ficha de notificação individual de violência interpessoal/autoprovocada, proposta pelo Ministério da Saúde, recebeu novos campos de preenchimento que permitem identificar ocorrências de violência contra a população LGBTQIA+. Foram acrescidas informações tais como nome social, orientação sexual, identidade de gênero e motivação da violência (6).

A obtenção de dados oriundos da notificação é fundamental para a formulação de políticas públicas destinadas a combater as diversas formas de violência. Devido a estigmatização, preconceitos estruturais e heteronormatizações sociais, poucas são as pesquisas que têm como foco avaliar as condições e a necessidades de saúde da população LGBTQIA+, o que dificulta inferir a real magnitude das condições enfrentadas por esses indivíduos (3). 

Ademais, os poucos estudos que aferem os episódios de violência contra a comunidade queer apresentam recortes para locais e curtos períodos de tempo, e analisam os membros da comunidade como um todo (2,7-11). É importante conduzir estudos específicos para os transgêneros, os quais são indivíduos marginalizados e apresentam mais chances de sofrer episódios de violência, crimes de ódio e privação aos serviços de saúde (12). Desse modo, o objetivo deste estudo foi caracterizar as notificações de violência contra travestis e transexuais, no Brasil, no período de 2015 a 2022.

## Métodos

### Delineamento

Trata-se de um estudo descritivo, de série temporal, conduzido segundo as orientações da declaração Reporting of Studies Conducted Using Observational Routinely Collected Data (13).

### Contexto

Na categorização dos tipos de violência, destacam-se a violência autoprovocada e a violência interpessoal, e ambas têm grande impacto nas condições de saúde e na qualidade de vida da população. Assim, o Ministério da Saúde orienta a notificação de todo caso suspeito ou confirmado de violência (5). 

### Participantes

Consideraram-se elegíveis para o estudo as notificações de violência interpessoal/autoprovocada entre pessoas transexuais e travestis.

### Variáveis 

As variáveis selecionadas fazem parte das fichas de notificação de violência interpessoal/autoprovocada, a saber: Unidades Federativas agrupados por regiões brasileiras (Norte, Nordeste, Centro-Oeste, Sudeste e Sul); raça/cor da pele; idade, categorizada em adolescente (de 11 a 17 anos), jovem (de 18 a 24 anos), adulto (de 25 a 59 anos) e idoso (60 anos ou mais) (16,17); identidade de gênero; orientação sexual; local de ocorrência; recorrência da violência; lesão autoprovocada; motivo da violência; tipo de violência; modalidade da violência sexual; número de agressores envolvidos; vínculo/grau de parentesco do(a) agressor(a) com a vítima; e ano de ocorrência da violência (2015 a 2022).

### Fontes de dados e mensuração

Os dados secundários são provenientes do Sistema de Vigilância de violência interpessoal e autoprovocada do Sistema de Informação de Agravos de Notificação (VIVA/Sinan), do Departamento de Informática do Sistema Único de Saúde do Brasil (DATASUS), que consolida as fichas de notificação e de investigação de casos de agravos e doenças estabelecidas como notificações compulsórias (14).

### Tamanho do estudo

O recorte temporal da descrição dos dados foi de 2015 a 2022. Esse período foi selecionado devido à inclusão dos campos de identificação da identidade de gênero e orientação sexual, em 2014, nas fichas de notificação compulsória (6). Dados referentes ao ano 2023 não foram incluídos, por não estarem completamente disponíveis na época da coleta de dados desta pesquisa.

### Acesso aos dados e métodos de limpeza

Os dados foram acessados em janeiro de 2024, por meio do software Tab para Windows (TabWin), disponível online para download no site do DATASUS (14). Foi realizada a importação de arquivos, que posteriormente foram agregados em planilhas no software Microsoft Office Excel 2013. Para identificação da população-alvo do estudo, foram excluídos casos em que o campo “identidade de gênero” estivesse preenchido “não se aplica” ou “ignorado”, ou estivesse em “branco”. Selecionaram-se casos em que as vítimas foram identificadas como “travestis”, “mulheres transexuais” e “homens transexuais”. Após exclusão das notificações sem o preenchimento adequado, analisaram-se a incongruência dos casos e os campos preenchidos incorretamente ou com divergência de informações (15). Casos atípicos foram excluídos, a partir da análise descritiva, com tabelas de contingência e outliers (por exemplo, indivíduos com meses de idade e transgêneros, e indivíduos do sexo masculino quando selecionado o campo gestante). Notificações de vítimas com idade inferior a 11 anos também foram excluídas.

### Métodos estatísticos

Os dados foram organizados em planilhas do Excel e previamente conferidos por dois pesquisadores treinados. Posteriormente, foram inseridos no Statistical Package for the Social Sciences 27.0, tendo sido conduzida a análise descritiva com frequências absolutas (n) e relativas (%). 

## Resultados

No recorte temporal, selecionaram-se 37.104 notificações de violência interpessoal/autoprovocada contra indivíduos travestis e transexuais. Mulheres transexuais (66,1%), heterossexuais (54,1%), adultos (49,5%) e indivíduos de raça/cor da pele parda (44,5%) foram os grupos que predominantemente sofreram algum tipo de episódio violento, com notificação entre 2015 a 2022 (Tabela 1). 

**Tabela 1:** Perfil das vítimas de violência interpessoal/autoprovocada praticada contra travestis e transexuais. Brasil, 2015-2022 (n=37.104)

Variável	n (%)
**Identidade de gênero**	
Travesti	5.241 (14,1)
Mulher transexual	24.522 (66,1)
Homem transexual	7.341 (19,8)
**Orientação sexual**	
Heterossexual	20.069 (54,1)
Homossexual	10.421 (28,1)
Bissexual	1.112 (3,0)
Ignorado/em branco	5.502 (14,8)
**Faixa etária** (anos)	
Adolescente (11-17)	6.299 (17,0)
Jovem (18-24)	10.548 (28,4
Adulto (25-59)	18.367 (49,5)
Idoso (60 ou mais)	1.890 (5,1)
**Raça/cor da pele**	
Branca	14.148 (38,1)
Preta	3.710 (10,0)
Amarela	315 (0,8)
Parda	16.522 (44,5)
Indígena	455 (1,2)
Ignorado/em branco	1.956 (5,3)

A distribuição de notificações em relação ao tempo, segundo a identidade de gênero, apresenta o maior quantitativo de notificações de 2015 a 2022 para mulheres transexuais (n=24.522), sendo que, de 2018 a 2019, nota-se tendência estacionária nas notificações (n=3.153 e n=3.195), que caem em 2020 (n=2.752) e retornam com aumento importante em 2022 (n=4.304), conforme Figura 1. O aumento percentual entre os anos de 2015 e 2022 foi maior entre travestis (+182,0%); para mulheres transexuais, foi de 99,0%, e para homens transexuais foi de 149,0%.

**Figura 1:** Tendência temporal das notificações de violência interpessoal/autoprovocada contra transexuais e travestis. Brasil, 2015-2022 (n=37.104)

A maioria dos casos notificados ocorreu na residência das vítimas (64,0%), e os tipos de violência mais prevalentes foram violência física (61,7%), autoprovocada (30,1%), seguida pela psicológica/moral (22,9%). Dos casos notificados, 40,7% eram episódios recorrentes. A LGBTfobia constituiu-se como motivo do episódio violento em 6,2% dos casos (Tabela 2). 

**Tabela 2:** Caracterização da violência praticada contra travestis e transexuais. Brasil, 2015-2022 (n=37.104)

Variável	n (%)
**Local de ocorrência**	
Residência	23.748 (64,0)
Habitação coletiva	592 (1,6)
Escola	512 (1,4)
Local de prática esportiva	140 (0,4)
Bar ou similar	1.273 (3,4)
Via pública	6.780 (18,4)
Comércio/serviços	578 (1,6)
Indústrias/construção	80 (0,2)
Outros	1.743 (4,7)
Ignorado/em branco	1.658 (4,4)
**Episódio recorrente**	
Sim	15.107 (40,7)
Não	17.359 (46,8)
Ignorado/em branco	4.638 (11,5)
**Violência autoprovocada**	
Sim	11.176 (30,1)
Não	24.134 (65,0)
Ignorado/em branco	1.794 (4,9)
**Motivo da violência**	
Sexismo	3.092 (8,3)
Homofobia/lesbofobia/bifobia/transfobia	2.312 (6,2)
Racismo	40 (0,1)
Intolerância religiosa	45 (0,1)
Xenofobia	39 (0,1)
Conflito geracional	4.952 (13,3)
Situação de rua	1.419 (3,8)
Deficiência	366 (1,0)
Outros	10.257 (27,6)
Não se aplica	14.134 (38,1)
**Tipo de violência**	
Física	25.992 (61,7)
Psicológica/moral	9.648 (22,9)
Tortura	1.400 (3,3)
Sexual	3.396 (8,1)
Tráfico de seres humanos	23 (0,1)
Financeira/econômica	557 (1,3)
Negligência/abandono	993 (2,4)
Intervenção legal	86 (0,2)
**Modalidade da violência sexual**	
Assédio sexual	793 (22,8)
Estupro	2.537 (73,1)
Exploração sexual	141 (4,1)

O(a) provável autor(a) dos episódios se trata da própria vítima (29,9%), seguido pelo cônjuge (15,7%) e de conhecido(a)/amigo(a) (13,3%). A suspeita do uso de álcool pelo(a) autor(a) do crime foi notificada em 31,6% das notificações (Tabela 3). 

**Tabela 3:** Perfil do(a) provável autor(a) da violência praticada contra travestis e transexuais. Brasil, 2015 a 2022 (n=37.104)

Variável	n (%)
Número de envolvidos	
Um	26.857 (72,4)
Dois ou mais	8.010 (21,6)
Ignorado/em branco	2.237 (6,0)
Vínculo/grau de parentesco com a vítima	
Própria pessoa	10.509 (29,9)
Pai	986 (2,8)
Mãe	910 (2,6)
Padrasto	479 (1,4)
Madrasta	57 (0,2)
Cônjuge	5.501 (15,7)
Ex-cônjuge	1.790 (5,1)
Namorado(a)	1.448 (4,1)
Ex-namorado(a)	634 (1,8)
Filho(a)	991 (2,8)
Irmão(ã)	1.091 (3,1)
Conhecido(a)/amigo(a)	4.687 (13,3)
Cuidador(a)	101 (0,3)
Patrão(ao)/chefe	136 (0,4)
Pessoa com relação institucional	246 (0,7)
Policial/agente da lei	350 (1,0)
Desconhecido(a)	5.226 (14,9)

Quanto à série histórica por regiões brasileiras, foi identificada a tendência crescente no número de notificações no período de 2015-2022, sendo o maior número de casos nesse período na região Sudeste (n=18.666), seguida do Nordeste (n=7.052), Sul (n=5.549), Centro-Oeste (n=3.064) e Norte (n=2.548) (Figura 2). As regiões Sudeste e Centro-Oeste apresentaram aumento de 155,0% das notificações entre 2015 e 2022; a região Sul teve aumento de 81,0%; o Norte, de 72,0%; e o Nordeste, de 69,0%. 

**Figura 2:** Tendência temporal das notificações de violência interpessoal/autoprovocada contra transexuais e travestis por região. Brasil, 2015-2022 (n=37.104)

## Discussão

A pesquisa contribui para o Sistema Único de Saúde (SUS) ao apresentar dados sobre a violência contra travestis e transexuais no Brasil. Constatou-se o crescimento de notificações de agressões dirigidas às pessoas transgêneros e travestis, registradas no Sinan, em todas as regiões do Brasil, principalmente na região Sudeste. É importante destacar que a maioria destas notificações foram identificadas para mulheres transexuais. Este dado enfatiza a constatação de que, ao divergirem das normas sociais estabelecidas, as manifestações de estranhamento em relação às expressões de sexualidade e gênero podem desencadear atos violentos (9). 

Quando mulheres trans enfrentam exclusões devido à sua identidade de gênero, seja em ambientes públicos ou privados, isso amplifica a criação de cenários marginalizados, contribuindo para experiências de abandono educacional e para condições socioeconômicas precárias (18).

Evidenciou-se que as faixas etárias mais acometidas pela violência foram jovens e adultos. As idades predominantes descritas na literatura variam entre 25 e 59 anos (2). Porém, para pessoas transgênero, as ocorrências entre mulheres transexuais foram mais comuns em todas as categorias de idade a partir da adolescência, com proporção de 37,0% entre adolescentes, 36,3% entre idosas e 31,8% entre pessoas adultas. As vítimas costumam ser pardas ou brancas (9). Importa salientar que a descrição da raça/cor da pele, na ficha de notificação compulsória, é baseada na autodeclaração (11,19).

Identificou-se que os principais cenários em que ocorreram as agressões contra indivíduos LGBTQIA+ foram residências e vias públicas. Nos casos que envolvem violência no contexto privado, entende-se que a unidade familiar ou íntima não atua como uma rede de suporte e proteção, agravando os efeitos dos danos provocados pela discriminação social vivenciada nos espaços públicos. Um estudo etnográfico com pessoas trans, realizado em Santa Maria/Rio Grande do Sul, em 2012, destacou que o lar é o ambiente onde se manifestam precocemente comportamentos violentos de preconceito, discriminação e agressões físicas, resultando, por vezes, na expulsão do domicílio (20,21).

Os tipos de violência mais prevalentes foram a física, a psicológica e a autoprovocada, além da violência sexual, classificada como estupro. Em estudo transversal desenvolvido por meio da análise das notificações de casos suspeitos ou confirmados de violência interpessoal, no período de 2016 a 2020, na cidade de São Paulo, o tipo de violência mais registrado foi a física, com 76,3% (n=3.683), acompanhada da psicológica/moral, com 32,6% (n=1.576), e da sexual, com 17,7% (n=854). Quase todas as notificações relativas às pessoas transgênero apresentavam antecedentes de abuso psicológico, e mais de 80,0% dessas pessoas já haviam sido vítimas de violência física ou sexual (11,22). 

Destaca-se, ainda, que a violência sexual, quando direcionada às pessoas que se identificam como LGBTQIA+, pode adquirir uma conotação distinta, sendo empregada como forma de castigo e humilhação às vítimas, por serem quem são. Dessa forma, a estigmatização e a vergonha geram a exclusão social que impacta, entre outros aspectos, o acesso/busca à assistência em saúde e a dificuldade de alcançar os serviços (23). A violência psicológica pode ser definida como agressões à identidade e outras qualidades essenciais do indivíduo, como a autoestima, tanto por ação quanto por omissão. Com essa perspectiva, podemos relacioná-la às situações enfrentadas diariamente por pessoas transexuais, nas quais suas identidades são ignoradas e não respeitadas em diversos espaços sociais (24).

Quase um terço das notificações analisadas referem-se à violência autoprovocada. A comunidade LGBTQIA+ enfrenta, particularmente, uma série de violências que se sobrepõem, afetando a saúde e contribuindo para o sofrimento e o adoecimento (8). Evidências apontam que a violência autoprovocada apresenta taxas elevadas entre membros da comunidade LGBTQIA+ quando comparadas com indivíduos heterossexuais. Ressalta-se que pessoas transexuais enfrentam um índice elevado de risco de suicídio em comparação com a população em geral (2).

Ao se analisarem os fatores de reincidência, orientação sexual e identidade de gênero por faixas etárias e compará-los com a população em geral, constata-se que os adultos jovens homossexuais têm um risco 87,0% maior e os adultos transexuais/travestis um risco 66,0% maior de apresentarem violência autoprovocada (10). Considerando-se que a residência foi o local onde mais ocorreram situações de violência, a prática de lesões autoinfligidas pode estar associada à negação da orientação sexual e da identidade de gênero das pessoas dentro do contexto das relações familiares (9).

Essa realidade se estende a outros setores, nos quais a aceitação e a oportunidade de emprego são desafiadoras, destacando-se, consequentemente, a prevalência do trabalho informal. Como resultado, muitas pessoas transexuais e travestis encontram-se no trabalho ligado à prostituição, reconhecido como um ambiente propício a diversas formas de violência (25). Nessa perspectiva, os marcadores relacionados ao gênero e à diversidade sexual são reconhecidos como componentes dos determinantes sociais do processo saúde e doença, na instituição da Política Nacional de Saúde Integral LGBT, tendo em vista a necessidade de ações de equidade no âmbito do SUS junto a esta população (9).

A falta de garantia de direitos à população transexual e a violência naturalizada contra esse público tendem a persistir, sendo necessária a busca ativa contínua de soluções para erradicação da violência. É urgente dar continuidade à inclusão social das pessoas trans nas atividades institucionais, por parcerias nas lutas cotidianas, tais como a implementação de legislações no sentido de prevenir a violência, investigar e responsabilizar os criminosos. Ademais, devem-se afastar os discursos violentos e transfóbicos, e repudiar os grupos trans excludentes e as tentativas de perseguição, seja pelo silenciamento ou por atos de violência (26).

Destaca-se, entre as notificações de violência, a perda de informações para certas variáveis, o que se constitui em uma limitação do estudo. Deve-se considerar, também, a possibilidade de o número de casos de violência contra o grupo estudado ser maior que o apresentado, por ausência de notificação das ocorrências. Como fortaleza do presente estudo, todos os casos válidos para o público-alvo, travestis e transexuais, no Brasil, foram considerados, na perspectiva dos oito anos da série temporal. 

A análise das notificações revela um cenário alarmante de violência contra travestis e transexuais no Brasil, entre 2015 e 2022. Identifica-se a predominância de mulheres transexuais como principais vítimas, juntamente com a alta ocorrência de episódios em residências. A repetição dos episódios de violência deu-se para quase metade das notificações, e uma das motivações para o ato violento foi a LGBTfobia, embora a violência autoprovocada também seja frequente. Sobre a tendência temporal das notificações por macrorregiões nacionais, identificaram-se maiores números de casos nas regiões Sudeste e Nordeste; no entanto, a região Centro-Oeste se iguala ao Sudeste no que diz respeito ao aumento percentual das notificações.

Os dados desta pesquisa auxiliam para a compreensão, pelo Estado brasileiro, acerca do problema da LGBTfobia, bem como para o embasamento da importância de políticas públicas no combate à violência contra a população transexual e travesti e na promoção do acolhimento e da equidade nesse segmento populacional. 

## Data Availability

O banco de dados e os códigos de análise utilizados na pesquisa estão disponíveis em http://tabnet.datasus.gov.br/cgi/deftohtm.exe?sinannet/cnv/violebr.def.
